# Statistical insights into the reaction of fluorine atoms with silicon

**DOI:** 10.1038/s41598-020-70432-0

**Published:** 2020-08-12

**Authors:** Rimantas Knizikevičius

**Affiliations:** Department of Physics, Kaunas University of Technology, 73 K. Donelaičio St., 44249 Kaunas, Lithuania

**Keywords:** Chemical physics, Surface chemistry

## Abstract

The dependences of silicon etching rate on the concentration of F atoms are investigated theoretically. The nonlinear regression analysis of the experimental data indicates that the reaction of F atoms with silicon is 2nd overall order reaction. The relationship between overall reaction order and kinetic reaction order is established using the etching rate equation. It is found that kinetic reaction order monotonically decreases with the increase in concentration of F atoms due to the increased surface coverage. Surface passivation by the reaction products is not observed under the investigated experimental conditions.

## Introduction

Scanning tunnelling microscopy (STM) is widely used to analyse chemical reactions taking place on the silicon surface. STM enabled to measure the etching rate dependences on the initial concentration of SiCl, SiBr, and SiI radicals^1,2^. During the experiments, silicon substrates were dosed with molecular halogens at room temperature in order to obtain the desired initial concentration. Subsequently, silicon substrates were heated because chemical reactions between the chemisorbed radicals occur only at elevated temperatures. STM provided useful information about the reaction and desorption pathways^3^ and confirmed that silicon dihalides are the final reaction products. The same experimental procedure is used to investigate chemical reaction between SiF radicals on the silicon surface. The etching rate is measured only at low initial concentration of SiF radicals because fluorine atoms due to small atomic radius penetrate into the silicon lattice^4,5^. The experimental measurements^6^ shown that at temperature 825 K planar removal of silicon atoms occurs together with multilayer pitting, which result in the increased surface roughness.

Steady-state reaction of F atoms with silicon is investigated using large array of diagnostic techniques^7^. The most common observations are following:

- SiF_4_ molecules detected by mass spectrometry of exhaust species^8^;
- ${\text{SiF}}_{{\text{x}}} \;\left( {{\text{x}} \le 3} \right)$ radicals found on the etched Si surface using X-ray photoemission spectroscopy^9^;
- SiF_2_ molecules detected by chemiluminescence^10^ and laser-induced fluorescence spectroscopy^11^;
- SiF_2_ molecules polymerize on the surface^12^.
- SiF radicals passivate the Si surface^13^;

The experimental measurements are subsequently analysed using theoretical models. Simplest theoretical models successfully describe specific phenomena^14^, while simulators provide more complete view of the etching process.

The overall reaction order is determined using isothermal dependences of silicon etching rate on the concentration of F atoms. The mean lifetime of F atoms under most common plasma processing conditions is several microseconds^15^. Within that time F atoms must diffuse from plasma to the silicon substrate and chemisorb on the surface. The flow of F atoms always has other species admixed. This affects surface coverage and changes reactivity of silicon substrates^16^. The number of artefacts is significantly reduced by using F_2_ plasma as the source of fluorine atoms. In this case, special design of the experimental setup is required to handle highly reactive F atoms and F_2_ gas. Only two research groups successfully performed the experiment:

1. in the work^17^, fluorine atoms were produced by the dissociation of 98.5% pure F_2_ gas using 14 MHz discharge in an alumina tube 50 cm upstream of the reaction cell. The flow rate of F_2_ gas was kept constant at 44 sccm, while concentration of F atoms varied from $1.0 \times 10^{15} \;{\text{cm}}^{ - 3}$ at 3.8 W to $5.1 \times 10^{15} \;{\text{cm}}^{ - 3}$ at 78 W discharge power. The concentration of F atoms was measured by gas-phase titration with Cl_2_ gas. Si(100) substrates were bonded to the end of aluminium rod with epoxy and positioned in the aluminium reaction cell. The reaction cell was thermally insulated, while the sample-holding rod was heated or cooled, depending on the desired temperature. The etching rate was calculated by dividing the etched 2-mm-wide trench depth by the etching time.
2. in the work^18^, fluorine atoms were produced by the dissociation of 99% pure F_2_ gas using 2.45 GHz discharge in an alumina tube outside the reaction chamber. The flow rate of F_2_ gas was kept constant at 8 sccm, while pumping speed was adjusted by the throttle valve. Concentration of F atoms varied from $1.1 \times 10^{12} \;{\text{cm}}^{ - 3}$ at 150 W to $3.6 \times 10^{14} \;{\text{cm}}^{ - 3}$ at 220 W discharge power. The concentration of F atoms was measured by gas-phase titration with H_2_ gas. Si(100) substrates were placed in the stainless steel reaction chamber. The temperature of silicon substrates was kept constant at 300 K. The etching rate was calculated by dividing the etched trench depth by the etching time.

The experimental scientists described steady-state etching rate using the empirical equation ${\text{V}} = \upvarepsilon^{\dag } \left[ {\text{F}} \right]^{\upgamma }$, where $\upvarepsilon^{\dag }$ is the rate constant and γ is the kinetic reaction order. In the proposed equation, kinetic reaction order describes how silicon etching rate depends on the concentration of F atoms. US-based research group^17^ assumed that the measured dependence is linear, and the kinetic reaction order is equal to 1. Japan-based research group^18^ used plot $\log_{10} \,{\text{V}}$ versus $\log_{10}\, \left[ {\text{F}} \right]$ to determine the kinetic reaction order. The linear plot provided the same averaged value of kinetic reaction order. However, the method used to determine kinetic reaction order is unreliable in the presence of intermediate reaction products. It is important to note that both research groups wrongly assumed that formed reaction products immediately desorb. As a result, the etching process was described using single activation energy, derived from the rate constant $\upvarepsilon^{\dag }$. At that time, the elementary processes were well documented^19^ but their activation energies in most cases were unknown. This resulted in the widespread assumption that the etching process requires insignificant activation energy. Recent theoretical calculations^20,21^ indicate that desorption process must be included in the description of the experimental data because of the high energetic barrier.

In this work, the isothermal dependences of silicon etching rate on the concentration of F atoms are reanalysed using the chemical kinetics. The overall reaction order is determined using nonlinear regression analysis of the experimental data. The etching rate equation enabled to establish relationship between overall reaction order and kinetic reaction order. It is found that kinetic reaction order equals to the Si surface fraction not covered with adsorbate.

## Theory

The enthalpy changes during the reactions of F atoms with silicon on the surface and silicon fluorides in the gas phase are presented in Table 1. In order to derive the overall reaction order, the following reactions of F atoms with silicon substrate are considered:

1 $${\text{Si}}\left( {\text{s}} \right) + {\text{nF}}\left( {\text{g}} \right) \to {\text{SiF}}_{{\text{n}}} \left( {\text{a}} \right),$$

where $1 \le {\text{n}} \le 4$ is the partial reaction order for F atoms. The overall reaction order is equal to ${\text{m}} = {\text{n}} + 1$. The reaction rate is described using transition-state theory by the reaction rate constant k_r_. The mean reaction time is equal to $\uptau_{{\text{r}}} = {\text{k}}_{{\text{r}}}^{ - 1} {\text{p}}_{{\text{F}}}^{ - {\text{n}}}$, where p_F_ is the partial pressure of F atoms. The etching process takes place when the reaction products desorb. The desorption rate constant according to the transition-state theory is equal to

2 $$\upomega = \frac{{\text{kT}}}{{\text{h}}}\exp \left( { - \frac{{{\text{E}}_{{\text{d}}} }}{{\text{kT}}}} \right),$$

where h is the Planck constant, k is the Boltzmann constant, T is the temperature, and E_d_ is the desorption activation energy. The mean desorption time is equal to $\uptau_{{\text{d}}} = \upomega^{ - 1}$. The relative error of desorption rate constant is equal to

3 $$\frac{{\Delta \upomega} }{{\upomega} } = \left( {1 + \frac{{{\text{E}}_{{\text{d}}} }}{{\text{kT}}}} \right)\frac{{\Delta}{\text{T}}}{{\text{T}}} + \frac{{{\Delta} {\text{E}}_{{\text{d}}} }}{{\text{kT}}}.$$

When the substrate temperature is determined accurately, the absolute error of desorption activation energy reaches maximum value

4a $${\Delta} {\text{E}}_{{\text{d}},\;\max } = {\text{kT}}\frac{{\Delta \upomega} }{{\upomega} }.$$

Otherwise, when the desorption activation energy of the reaction product is determined accurately, the absolute error of temperature reaches maximum value

4b $${\Delta} {\text{T}}_{\max } = \left( {\frac{{{\text{kT}}^{2} }}{{{\text{E}}_{{\text{d}}} + {\text{kT}}}}} \right)\frac{{\Delta \upomega} }{{\upomega} }.$$

**Table 1:** The reaction enthalpies, measured in electronvolts, at standard conditions.

Reactant	Reaction product			
	SiF(g)	SiF_2_(g)	SiF_3_(g)	SiF_4_(g)
Si(s)	− 1.03	− 7.74	− 13.72	− 20.03
SiF(g)	0	− 6.71	− 12.69	− 19.00
SiF_2_(g)	–	0	− 5.98	− 12.29
SiF_3_(g)	–	–	0	− 6.31

SiF_n_ species are included in the adsorbed layer of one-monolayer thickness. The relative concentration of SiF_n_ species in the adsorbed layer is equal to ${\text{c}} = {{\left[ {{\text{SiF}}_{{\text{n}}} } \right]} \mathord{\left/ {\vphantom {{\left[ {{\text{SiF}}_{{\text{n}}} } \right]} {\text{C}}}} \right. \kern-\nulldelimiterspace} {\text{C}}}$, where ${\text{C}} = 6.78 \times 10^{18} \;{\text{m}}^{ - 2}$ is the planar density of Si(100) substrates. The following differential equation includes rate expressions of the processes mentioned earlier and describes the concentration kinetics in the adsorbed layer:

5 $$\frac{{\text{dc}}}{{{\text{dt}}}} = \upbeta {\text{k}}_{{\text{r}}}\, {\text{p}}_{{\text{F}}}^{{\text{n}}} - \upomega {\text{c}},$$

where $\upbeta = 1 - {\Theta}$ is the surface fraction not covered with adsorbate and ${\Theta} = {\text{c}}$ is the surface coverage. The steady-state concentration of SiF_n_ species is equal to

6 $${\text{c}}_{{\text{st}}} = {{{\text{k}}_{{\text{r}}}\,{\text{p}}_{{\text{F}}}^{{\text{n}}} } \mathord{\left/ {\vphantom {{{\text{k}}_{{\text{r}}} \,{\text{p}}_{{\text{F}}}^{{\text{n}}} } {\left( {{\text{k}}_{{\text{r}}} {\text{p}}_{{\text{F}}}^{{\text{n}}} + \upomega } \right)}}} \right. \kern-\nulldelimiterspace} {\left( {{\text{k}}_{{\text{r}}} {\text{p}}_{{\text{F}}}^{{\text{n}}} + \upomega } \right)}}.$$

The steady-state etching rate, which is equal to the desorption rate of formed SiF_n_ species, is calculated using the following equation:

7 $${\text{V}}_{{\text{st}}} = \left( {\uptau_{{\text{r}}} + \uptau_{{\text{d}}} } \right)^{ - 1} = \upomega {\text{c}}_{{\text{st}}} = \frac{{{\text{k}}_{{\text{r}}}\,{\text{p}}_{{\text{F}}}^{{\text{n}}} \upomega }}{{{\text{k}}_{{\text{r}}} {\text{p}}_{{\text{F}}}^{{\text{n}}} + \upomega }}.$$

Kinetic reaction order for the above mentioned chemical reactions under steady-state conditions is calculated using the following equation:

8 $$\upgamma = \frac{{{\text{dV}}_{{\text{st}}} }}{{{\text{dp}}_{{\text{F}}} }} \times \frac{{{\text{p}}_{{\text{F}}} }}{{{\text{V}}_{{\text{st}}} }} = \frac{{\text{n}}\upomega }{{{\text{k}}_{{\text{r}}} {\text{p}}_{{\text{F}}}^{{\text{n}}} + \upomega }}.$$

It is important to note that kinetic reaction order monotonically decreases with the increase in partial pressure of F atoms. Meanwhile, the overall reaction order does not depend on the partial pressure of F atoms.

## Results and discussion

**Figure 1:**
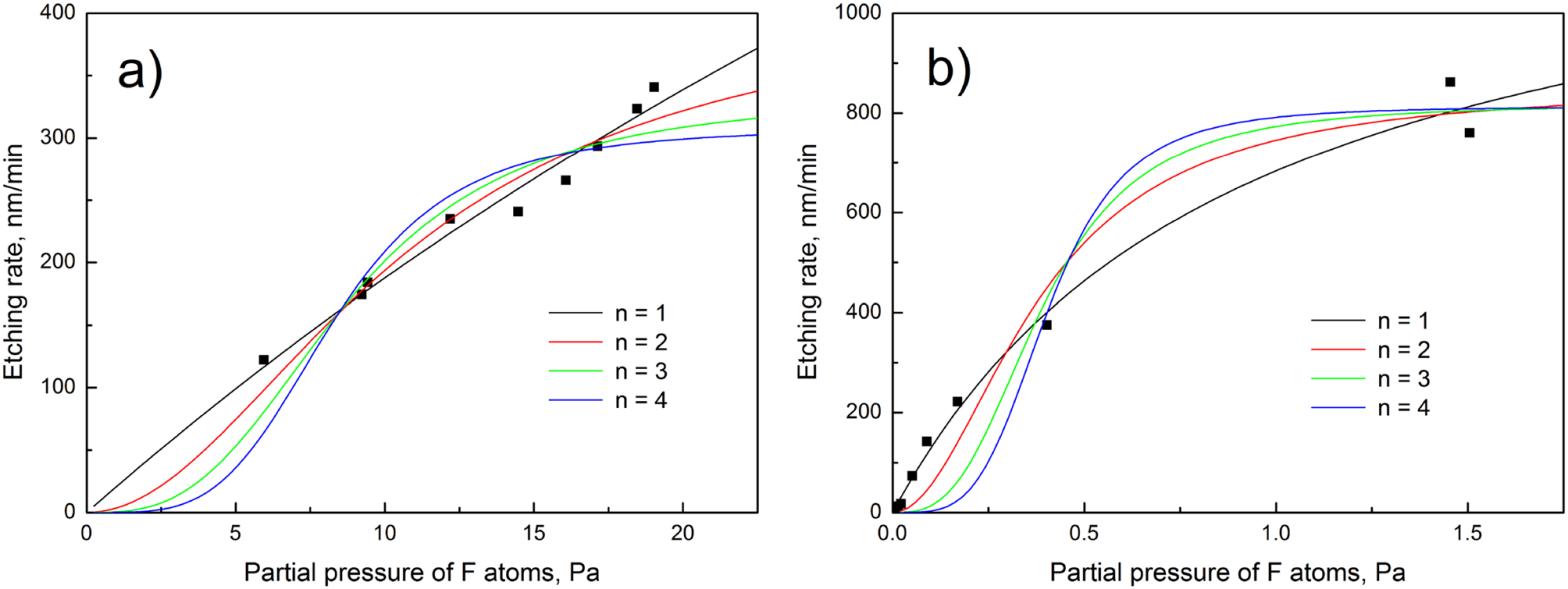
The experimental dependences of silicon etching rate on the partial pressure of F atoms fitted using Origin Pro software: (**a**) experiment^17^, (**b**) experiment^18^.

The reaction of fluorine atoms with silicon at constant temperature is investigated using the nonlinear regression of the experimental data. The experimental and theoretical dependences of silicon etching rate on the partial pressure of F atoms are shown in Fig. 1. In both cases, best quality fits are obtained using first partial reaction order for F atoms, see Table 2. Moreover, the values of R-square and adjusted R-square indicate that the theoretical model better fits the experimental data^18^. The goodness-of-fit parameters are thoroughly described in the work^22^. According to the chemical kinetics, the reaction of F atoms with silicon is 2nd overall order reaction. This means that SiF radicals are the final reaction product. However, SiF radicals tend to passivate the Si surface, and suppress the etching rate at high partial pressure of F atoms in the plasma^13^. The experiment was performed in the parallel-plate plasma deposition system because of high working pressure (up to 300 Pa). While, the theoretical results showed that the etching rate is equal to the desorption rate of formed SiF_2_ molecules. Let us adopt the theoretical results presented in the work^13^ and assume that SiF_2_ molecules prevail in the flux of desorbing reaction products. Chemiluminescence^23,24^ and laser-induced fluorescence^25,26^ spectroscopy methods show large amounts of SiF_2_ molecules in the flux of desorbing reaction products. SiF_2_ molecules are formed during the following reaction in the adsorbed layer:

9 $${\text{2SiF}}\left( {\text{a}} \right){\mathop{\longrightarrow}\limits^{{\text{SV}}/{\text{DB}}}}{\text{SiF}}_{2} \left( {\text{a}} \right) + {\text{Si}}\left( {\text{s}} \right).$$

where SV is the surface vacancy and DB is the dangling bond. The conversion of SiF radicals into SiF_2_ molecules in the presence of surface defects is confirmed experimentally^6^. Sophisticated experimental equipment is required in order to detect desorbing SiF_2_ molecules because they are stable only at elevated temperatures. At room temperature, SiF_2_ molecules, regardless of their relatively high mean lifetime in the gas phase^27^, are converted into SiF_4_ molecules. Therefore, simplest measurement methods such as mass spectrometry struggle to detect SiF_2_ molecules. The temperature-programmed desorption experiments indicate that mean lifetime of SiF_3_ radicals in the gas phase is much lower than that of SiF_2_ molecules^28^.

**Table 2:** The goodness-of-fit parameters obtained during the nonlinear regression analysis of the experimental data. Standard deviation (SD) is equal to the square root of reduced-chi-square.

n	Reduced Chi-Sqr	SD	R-square	Adj. R-square
**Experiment**^17^, ${\text{T}} = 296\;{\text{K}}$				
1	161.84	12.722	0.97298	0.96912
2	384.43	19.607	0.93581	0.92664
3	754.31	27.465	0.87405	0.85606
4	1,210.20	34.788	0.79793	0.76906
**Experiment**^18^, ${\text{T}} = 300\;{\text{K}}$				
1	905.20	30.087	0.99163	0.99070
2	3,492.51	59.097	0.96770	0.96411
3	6,365.95	79.787	0.94112	0.93457
4	7,879.57	88.767	0.92712	0.91902

The nonlinear regression analysis of the experimental data yields kinetic parameters associated with ${\text{Si}} + {\text{nF}} \to{\text{SiF}}_{{\text{n}}}$ reactions. The reaction and desorption rate constants, determined using different partial reaction orders for F atoms, are presented in Table 3. The reaction rate constants describe how silicon etching rate increases with the increase in partial pressure of F atoms. The reaction rate constants are determined accurately using first partial reaction order. However, the relative error of the reaction rate constants sharply increases with the increase in partial reaction order. The reaction activation energies are not calculated because high reaction activation entropy can lead to significant errors and misinterpretations. Desorption rate constants describe asymptotical approach of the etching rate to the saturation regime. The desorption rate constants are determined accurately except one at temperature 296 K using first partial reaction order for F atoms. In this particular case, the predicted dependence is nearly linear in the considered range of partial pressure of F atoms, and the fitting program yields large relative error of the desorption rate constant. The average desorption activation energy of the reaction product determined using first partial reaction order is equal to $\left( {0.640 \pm 0.007} \right)\,{\text{eV}}$.

**Table 3:** The kinetic parameters determined using different partial reaction orders for F atoms.

n	k_r_, Pa^−n^ s^−1^	k_r_, m^3n^ mol^−n^ s^−1^	${\Delta} {\text{k}}_{{\text{r}}} /{\text{k}}_{{\text{r}}}$	$\upomega ,\,{\text{s}}^{ - 1}$	${\Delta} \upomega /\upomega$	E_d_, eV	${\Delta} {\text{E}}_{{\text{d}},\;\max } ,\;{\text{eV}}$	${\Delta} {\text{T}}_{\max } ,\;{\text{K}}$
**Experiment**^17^, ${\text{T}} = 296\;{\text{K}}$								
1	1.290	3,174	0.09	106.4	0.47	0.632	0.012	6
2	2.234 × 10^−1^	1.353 × 10^6^	0.15	25.32	0.08	0.669	0.002	1
3	3.104 × 10^−2^	4.628 × 10^8^	0.23	20.48	0.06	0.674	0.002	1
4	3.980 × 10^−3^	1.461 × 10^11^	0.33	18.88	0.06	0.676	0.002	1
**Experiment**^18^, ${\text{T}} = 300\;{\text{K}}$								
1	88.10	2.198 × 10^5^	0.11	79.88	0.08	0.648	0.002	1
2	359.0	2.233 × 10^9^	0.26	52.35	0.06	0.659	0.002	1
3	844.5	1.310 × 10^11^	0.39	50.19	0.07	0.660	0.002	1
4	1833	7.097 × 10^16^	0.44	49.82	0.08	0.661	0.002	1

In work^29^, desorption activation energy of SiF_4_ molecules at temperature 296 K was evaluated using fourth partial reaction order for F atoms. It was determined that the desorption activation energy of SiF_4_ molecules is equal to $\left( {0.66 \pm 0.03} \right)\,{\text{eV}}$. The obtained theoretical results showed that desorption of the reaction product requires significant activation energy. However, the reaction product prevailing in the flux of desorbing species was not identified correctly. In work^20^, desorption activation energy of SiF_2_ molecules at temperature 376 K was evaluated using first partial reaction order for F_2_ molecules. It was determined that desorption activation energy of SiF_2_ molecules is equal to $\left( {0.815 \pm 0.010} \right)\,{\text{eV}}$. It is important to note that the difference between determined desorption activation energies is equal to 0.155 eV. This corresponds to the difference in the substrate temperature 1,800 K, which exceeds the silicon melting point at standard conditions. However, melting of the silicon substrates was not observed experimentally. The experimental data^19^ indicate that activation energies of elementary processes increase with the increase in temperature during chemical etching of silicon in F_2_ environment. At room temperature, diffusion of Si atoms on the surface is strongly anisotropic. Diffusion activation energy for Si atoms along dimer rows is much lower than that between dimer rows^30,31^. The reconstruction of silicon surface becomes pronounced at temperatures above 350 K. This phenomenon reduces diffusion of Si atoms along the dimer rows below the expected thermal values^32^, and increases the activation energies of elementary processes.

Let us consider the etching process using another statistical approach. The reaction constant shows how many Si atoms are removed from the surface by single F atom

10 $$\upvarepsilon = \frac{{{\Phi} \left( {{\text{SiF}}_{2} } \right)}}{2{\Phi} \left( {\text{F}} \right)} = \frac{{{\text{k}}_{{\text{r}}} \upomega {\text{C}}}}{{{\text{k}}_{{\text{r}}} \,{\text{p}}_{{\text{F}}} + \upomega }}\sqrt{\frac{\uppi {\text{m}}_{{\text{F}}} {\text{kT}}}{2}} ,$$

where ${\Phi} \left( {{\text{SiF}}_{2} } \right) = \upomega \left[ {{\text{SiF}}_{2} } \right]$ is the flux of desorbing SiF_2_ molecules, ${\Phi} \left( {\text{F}} \right) = {\text{p}}_{{\text{F}}} \left( {2{\uppi}{\text{m}}_{{\text{F}}} \,{\text{kT}}}\right)^{{ - {1 \mathord{\left/ {\vphantom {1 2}} \right. \kern-\nulldelimiterspace} 2}}}$ is the flux of F atoms to the silicon surface, and m_F_ is the mass of F atom. It is important to note that reaction constant depends on the partial pressure of F atoms. At extremely low pressure, the reaction constant reaches maximum value

11 $$\upvarepsilon_{0} = {\text{k}}_{{\text{r}}} \,{\text{C}}\sqrt {\frac{{\uppi {\text{m}}_{{\text{F}}}\, {\text{kT}}}}{2}} .$$

According to Eq. (6), the ratio ${\upvarepsilon \mathord{\left/ {\vphantom {\upvarepsilon {\varepsilon_{0} }}} \right. \kern-\nulldelimiterspace} {\upvarepsilon_{0} }}$ is equal to the surface fraction not covered with adsorbate

12 $$\frac{\upvarepsilon }{{\upvarepsilon_{0} }} = \frac{\upomega }{{{\text{k}}_{{\text{r}}} \,{\text{p}}_{{\text{F}}} + \upomega }} = \upbeta .$$

**Figure 2:**
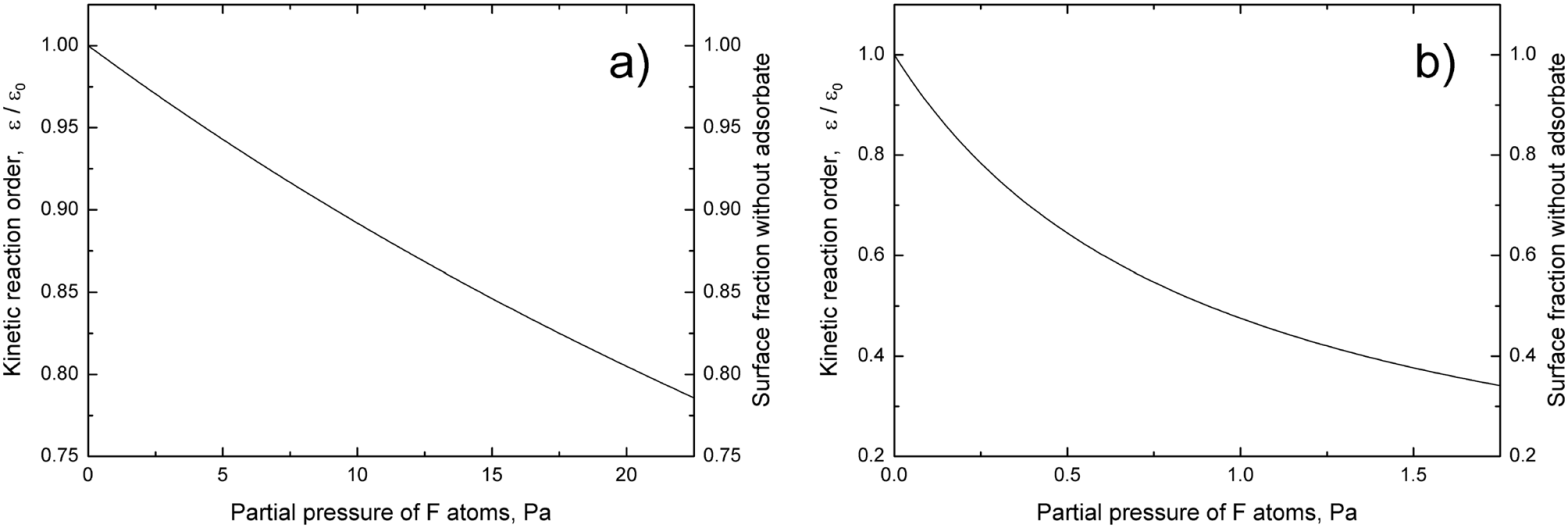
The theoretical dependences of kinetic reaction order, ratio ε/ε_0_, and surface area not covered with adsorbate on the partial pressure of F atoms: (**a**) experiment^17^, (**b**) experiment^18^.

The dependences of ratio ${\upvarepsilon \mathord{\left/ {\vphantom {\upvarepsilon {\upvarepsilon_{0} }}} \right. \kern-\nulldelimiterspace} {\upvarepsilon_{0} }}$ on the partial pressure of F atoms are shown in Fig. 2. The maximum value of reaction constant during the experiment^17^ is equal to $\upvarepsilon_{0} = 2.50 \times 10^{ - 4}$ (99.95% of F atoms incident to the atomically clean silicon surface are reflected). Meanwhile, the maximum value of reaction constant during the experiment^18^ is equal to $\upvarepsilon_{0} = 1.72 \times 10^{ - 2}$ (96.56% of F atoms incident to the atomically clean silicon surface are reflected). Although the experimental conditions are very similar, the reaction constants ε_0_ differ 69 times. The difference can be caused by measurement errors made during the gas phase titration and by exposure of silicon substrates to the plasma radiation with the subsequent increase in temperature^33,34^. The substrate temperature can also increase due to poor thermal contact between the substrate and the substrate holder^35^. Highest absolute error of temperature (about 6 K) is obtained during nonlinear regression of the experimental data^17^. However, the temperature uncertainty cannot explain the observed difference between the reaction constants.

Kinetic reaction order is calculated using first partial reaction order for F atoms. SiF radicals are formed rapidly in the adsorbed layer but subsequently tend to passivate the silicon surface. The surface passivation is described by negative values of the kinetic reaction order. According to the model, kinetic reaction order monotonically decreases with the increase in partial pressure of F atoms but negative values of the kinetic reaction order are not achieved. The theoretical dependences of kinetic reaction order on the partial pressure of F atoms, presented in Fig. 2, indicate that concentration of SiF radicals in the adsorbed layer is insufficient to passivate the surface. SiF radicals are converted into SiF_2_ molecules, which subsequently desorb. In the case of first partial reaction order for F atoms, the same equation describes:

- the dependence of kinetic reaction order on the partial pressure of F atoms;
- the dependence of ratio ${\upvarepsilon \mathord{\left/ {\vphantom {\upvarepsilon {\upvarepsilon_{0} }}} \right. \kern-\nulldelimiterspace} {\upvarepsilon_{0} }}$ on the partial pressure of F atoms;
- the dependence of surface fraction not covered with adsorbate on the partial pressure of F atoms.

**Figure 3:**
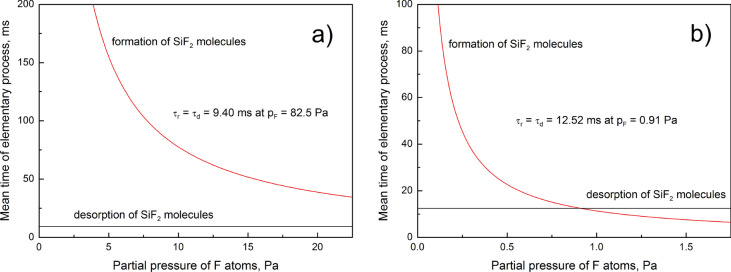
The theoretical dependences of mean times of elementary processes on the partial pressure of F atoms: (**a**) experiment^17^, (**b**) experiment^18^.

The mean reaction time and the mean desorption time provide an insight into the etching-rate limiting process. According to the model, mean reaction time reciprocally decreases with the increase in partial pressure of F atoms, while mean desorption time does not depend on the partial pressure of F atoms. The theoretical results, presented in Fig. 3, indicate that F atoms are much more reactive towards the silicon substrates during the experiment^18^. The difference can be caused by measurement errors made during the gas phase titration and by exposure of silicon substrates to the plasma radiation. However, higher mean desorption time of SiF_2_ molecules in the experiment^18^ indicates lower temperature of the silicon substrates (by about 3 K). This means that the exposure of silicon surface to the plasma radiation during the experiment^18^ can be neglected. The difference between the experiments^17,18^ is caused by the measurement errors made during the gas phase titration of F atoms. The statement is supported by the different ranges of partial pressure of F atoms used in the experiments.

**Figure 4:**
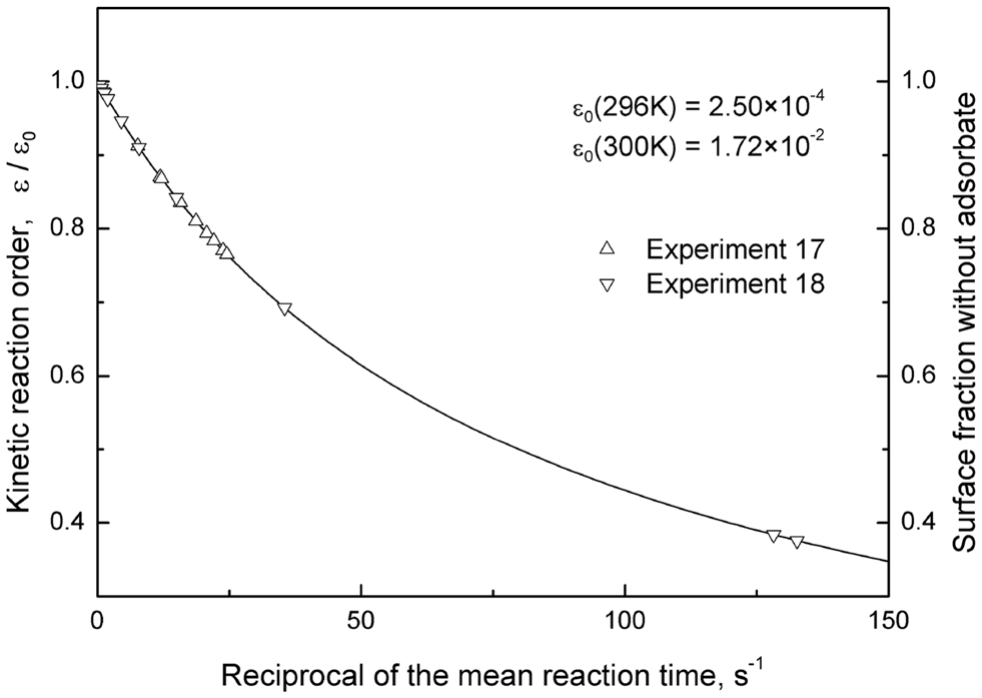
The dependences of kinetic reaction order, ratio ε/ε_0_, and surface area not covered with adsorbate on the reciprocal of the mean reaction time. The desorption rate constant $\upomega = 79.88\;{\text{s}}^{ - 1}$.

The experimental results^17,18^ converge into single curve at the same desorption rate constant. Kinetic reaction order, ratio ${\upvarepsilon \mathord{\left/ {\vphantom {\upvarepsilon {\upvarepsilon_{0} }}} \right. \kern-\nulldelimiterspace} {\upvarepsilon_{0} }}$, and surface fraction not covered with adsorbate are plotted versus reciprocal of the mean reaction time in Fig. 4. The data points shown on the curve indicate theoretically calculated values of three different parameters at experimentally measured partial pressure of F atoms. It is important to note that the last two data points corresponding to the kinetic reaction order of about 0.4 were intentionally omitted from the plot $\log_{10}\,{\text{V}}$ versus $\log_{10} \,\left[ {\text{F}} \right]$ in order to obtain the linear dependence by the Japan-based research group^18^. All other data points show that kinetic reaction order varies from 1 to approximately 0.7. This indicates that up to 30% of the silicon surface is covered by adsorbate. The coverage has little influence on the experimentally measured etching rate. Therefore, the experimental scientists tend to use term “pseudo-first kinetic reaction order”. In most cases, the term is shortened to the “pseudo-first reaction order”^25,36^, although this not correct from the view point of chemical kinetics.

Finally, the subtle difference between the proposed model and Langmuir adsorption model must be considered. Langmuir isotherms are obtained using the following assumptions^37^:

- surface with the adsorption sites is atomically flat;
- adsorption sites have the same adsorption activation energy;
- species adsorb only into an immobile state;
- surface coverage is one-monolayer thickness;
- adsorbed species are chemically inert.

The most notable difference between the proposed model and Langmuir adsorption model is that adsorbate diffuses on the surface. When single plasma component adsorbs on the surface, both models provide identical equations. The difference induced by the adsorbate diffusion becomes pronounced when two or more plasma components adsorb on the surface^38^. Additionally, the reaction between SiF radicals in the adsorbed layer was deduced by comparison of the obtained theoretical results with the experimental measurements.

## Conclusions

Chemisorption of F atoms on the silicon surface does not occur instantly. At room temperature, at least 96% of F atoms incident to the atomically clean silicon surface are reflected. The nonlinear regression analysis of the experimental data indicates that the reaction of F atoms with silicon is 2nd overall order reaction. The volatile reaction product is formed during the reaction ${\text{2SiF}}\left( {\text{a}} \right){\mathop{\longrightarrow}\limits^{{\text{SV}}/{\text{DB}}}}{\text{SiF}}_{2} \left( {\text{a}} \right) + {\text{Si}}\left( {\text{s}} \right)$ in the adsorbed layer. Kinetic reaction order monotonically decreases with the increase in partial pressure of F atoms due to the increased surface coverage. Surface passivation by SiF radicals is not observed under the investigated experimental conditions.
